# *LXN* deficiency regulates cytoskeleton remodelling by promoting proteolytic cleavage of Filamin A in vascular endothelial cells

**DOI:** 10.1111/jcmm.16685

**Published:** 2021-06-03

**Authors:** Guozhang He, Shuang Kan, Shaohua Xu, Xuchen Sun, Rong Li, Wei Shu, Ming Chen

**Affiliations:** ^1^ State Key Laboratory for the Chemistry and Molecular Engineering of Medicinal Resources School of Chemistry and Pharmacy Guangxi Normal University Guilin China; ^2^ Collaborative Innovation Center for Guangxi Ethnic Medicine Guangxi Normal University Guilin China; ^3^ College of Biotechnology Guilin Medical University Guilin China

**Keywords:** atherosclerosis, cytoskeleton remodelling, endothelial cells, laminar shear stress, LXN

## Abstract

Endothelial cells (ECs) respond to blood shear stress by changing their morphology is important for maintaining vascular homeostasis. Studies have documented a relationship between endothelial cell shape and the stress flow, and however, the mechanism underlying this cytoskeletal rearrangement due to shear stress remains uncertain. In this paper, we demonstrate that laminar shear stress (LSS) significantly reduces latexin (LXN) expression in ECs. By using siRNA and cell imaging, we demonstrated that LXN knockdown results in the morphologic change and F‐actin remodelling just like what LSS does in ECs. We further demonstrate that LXN interacts with Filamin A (FLNA) and regulates FLNA proteolytic cleavage and nuclei translocation. By constructing LXN^‐/‐^ mice and ApoE^‐/‐^LXN^‐/‐^ double knockout mice, we evaluated the effect of LXN knockout on aortic endothelium damage in mice. We found that LXN deficiency significantly improves vascular permeability, vasodilation and atherosclerosis in mice. Our findings provide confident evidence, for the first time, that LXN is a novel regulator for morphological maintenance of ECs, and LXN deficiency has a protective effect on vascular homeostasis. This provides new strategies and drug targets for the treatment of vascular diseases.

## 1 INTRODUCTION

Endothelial cell (EC) lines the luminal surface of blood vessels, and it is increasingly recognized that shape change keep EC in health states both in vitro and in vivo.^1^, ^2^ For example, it has been observed that the ECs in the arterial areas prone to early atherosclerosis are uniform polygonal or round, while in the anti‐atherosclerosis areas, the ECs are elongated.^3^, ^4^ ECs are continuously exposed to haemodynamic shear stress in vivo. When ECs sense shear stress, they change the cell shape, functions and gene expressions and involved in both physiologic and pathophysiologic vascular biology.^5^, ^6^ In this regard, two types of shear stress were reported in large vessel: one is laminar shear stress, and the other is disturbed shear stress; laminar shear stress (LSS) is vascular protective and founded in straight arterial regions (such as thoracic aorta). However, disturbed shear stress (DSS) is vascular harmful and founded in branched or curved regions (such as aortic arch). Interestingly, studies have shown that vascular permeability increases where the branches of blood vessels are located, along with an increased risk of atherosclerosis.^7^, ^8^, ^9^

Mechanotransduction by shear stress is complex. It can be roughly determined that shear stress activates various conduction pathways through membrane molecules, including calcium channel, G protein, tyrosine kinase receptor, adhesion protein and cytoskeleton.^10^, ^11^, ^12^, ^13^ Under shear stress, the most intuitive phenotype of ECs is morphological change: ECs undergo a transition from polygonal cobblestone‐like uniform sheets to uniform spindle‐like monolayer. This phenomenon is thought to be partly the result of F‐actin stress fibre induction,^14^, ^15^ and however, the mechanism underlying this cytoskeletal rearrangement due to shear stress is ill‐defined.

Latexin (LXN), a carboxypeptidase inhibitor with 222 amino acids in length, was first identified in the lateral neocortex of rats and acts as a marker of neurons in the lateral neocortex of developing rat brain.^16^, ^17^ Due to the characteristics of its protease inhibitor, it is thought that LXN might be involved in protein degradation and metabolism.^18^ LXN is also implicated in inflammation because it is expressed in macrophage and mast cells and can be induced by lipopolysaccharide.^19^ However, the function of LXN in ECs is unknown. In the present study, we found that laminar shear stress induces the down‐regulation of LXN in ECs, which results in the morphologic change and F‐actin remodelling just like what LSS does in ECs. Logically, we hypothesize LXN is a novel regulator that involves in the process of endothelial cell morphologic change and *LXN* deficiency has a protective effect on vascular homeostasis.

## 2 METHODS

### 2.1 Cells and reagents

HUVECs were purchased from ATCC and cultured in EBM‐2 medium (Lonza) supplemented with EGM‐2 BulletKit (Lonza). The following antibodies were used: mouse anti‐LXN (Sino Biological Inc, 1:1000 for WB), rabbit anti‐LXN (abcam, 1:1000 for WB, 1:100 for IF), mouse anti‐CD31 (abcam, 1:100 for IF), rabbit anti‐FAK (CST), anti‐β‐tubulin (CST), anti‐paxillin (CST), anti‐Arp2/3 (CST), anti‐α‐actinin (CST) and FLNA N‐terminal/ C‐terminal specific antibody (abcam, 1:1000 for WB, 1:100 for IF). Secondary antibodies for immunofluorescence were fluorescein‐5‐isothiocyanate (FITC)‐conjugated secondary antibodies (1:250 dilution, Invitrogen) and Tetramethylrhodamine isothiocyanate (TRITC)‐conjugated secondary antibodies (1:300 dilution; Invitrogen). Cell nuclei were stained with DAPI. SiRNA oligonucleotides was purchase from Sigma‐Aldrich (SASI_Hs01_00073945; SASI_Hs02_00325015), and a negative control siRNA (MISSION siRNA Universal Negative Control; Sigma‐Aldrich) was used for the transfection of HUVEC with Lipofectamine ^®^RNAiMAX transfecting reagent (Invitrogen) in serum‐free medium according to the manufacturer‘s recommendation.

### 2.2 Shear stress

Laminar shear stress was applied to cells by using parallel plate flow chambers (GlycoTech) set in series in a closed circulating system with 5% CO_2_ at 37℃. The parallel plate flow chamber comprised a polycarbonate plate attached to rectangular gaskets (Thickness: 0.010 in; Flow path width: 1.0 cm). HUVECs were cultured on a fibronectin coated slice. When cells grow to 90% confluence, attached the slice to flow chamber. The shear stress flow was initiated with EBM‐2 medium. Shear stress (15 dyne/cm^2^) was applied for indicated times. Cell morphology was demonstrated by Phase‐contrast photomicrograph.

### 2.3 Animal model

LXN^‐/‐^ mice were purchased from RIKEN BioResource centre (Japan)^20^ and maintained in 12 hours light/12 hours dark cycles with free access to food and water. ApoE^‐/‐^ mice (B6JNju‐Apoeem1Cd82Nju, C57BL/6 background) were purchased from GemPharmatech Co., Ltd. LXN^‐/‐^ mice were intercrossed with ApoE^‐/‐^ mice to generate ApoE^‐/‐^LXN^‐/‐^ mice. Mice genotype was determined by PCR using the primers as below: Pro‐F, 5′‐CGTTAGACTTTAAAATGCTCACTTTGGAAGCCCATACTC‐3′; Lax‐R, 5′‐CCTCCTTGCTGGCCTGCTGGACCGTCTGCACC‐3′; Apoe‐del82‐tF1: 5′‐TGCCTAGTCTCGGCTCTGAACTAC‐3′; Apoe‐del82‐tR1: 5′‐CAACCTGGGCTACACACTAATTGAG‐3′. For Miles assay, wild‐type (WT) and LXN^‐/‐^ mice were injected into the tail vein with 4 mL/kg of weight a 1% Evans blue dye solution (w/v in PBS). To determine microvessel vascular permeability, PBS or recombinant human VEGF‐165 (20 ng, 50 ng) and TNF‐α (10 ng, 30 ng) were intradermally injected into the shaved dorsal skin of the mice after 30 minutes Evans blue dye injection. After 20 minutes, the mice were sacrificed with CO_2_, and a small area of skin with blue spot was harvested and imaged. Evans Blue dye was extracted from the skin tissue by incubation with formamide, and the dye concentration was determined at 620 nm by using a spectrophotometer. For atherosclerosis mice model, 8‐week‐old ApoE^‐/‐^ and ApoE^‐/‐^LXN^‐/‐^ mice were fed with high‐fat diet (D12108C, Open Source Diets, Research Diets, Inc.) for 16 weeks. To determine the vasodilatation, thoracic aorta was isolated and mounted for tension measurements (DMT620 M, Danish Myo Technology). The aorta segments were stimulated by high potassium solution (KPSS) to a stable plateau stage and eluted to baseline by PSS solution at 37℃ for 5 minutes. The aorta segments were then equilibrated for 20 minutes prior to testing contractile capacity by exposure 1 µmol/L norepinephrine (NE). The vasodilatation ability of the vessels was then determined by exposure to acetylcholine. The entire aortas were isolated and were stained with HE or Oil Red O (Sigma‐Aldrich) for en face analysis. All procedures were conducted in accordance with the National Institutes of Health guidelines for the Care and Use of Laboratory Animals and approved by Institutional Animal Care and Use Committee at the Guangxi Normal University.

### 2.4 Real‐time quantitative PCR

Total RNAs were extracted from HUVECs by using miRNeasy Mini Kit (QIAGEN). qRT‐PCR was performed on cDNA from 200 ng of total RNA by using cDNA Synthesis kit and SYBR^®^ Green Master Mix Kit (Exqion). The primer sequences are described as follows: LXN, forward, 5′‐ACAGAACTACATCAACTACCAGC‐5′ and reverse, 5′‐GTGATACTTATGTCCTCTTCCTGG‐5′; VCAM1, forward, 5′‐TCTACGCTGACAATGAATCCTG‐5′ and reverse, 5′‐AGGGCCACTCAAATGAATCTC‐5′; CD36, forward, 5′‐TTGCAAGAAACAGGTGCTTAAC‐3′ and reverse, 5′‐GGTCTCCAACTGGCATTAGAATA‐3′; ANGPT2, forward, 5′‐ ATCAGGACACACCACGAATG‐3′ and reverse, 5′‐ CATCCTCACGTCGCTGAATAA‐3′; CPY1B1, forward, 5′‐CTGTCTTGGGCTACCACATT‐3′ and reverse, 5′‐GGATCAAAGTTCTCCGGGTTAG‐3′; THBD, forward, 5′‐ ACCAGGTCGTAGTTAGGGTAG‐3′ and reverse, 5′‐ACGTGGATGACTGCATACTG‐3′; NOS3, forward, 5′‐ CATCACCAGGAAGAAGACCTTTA‐3′ and reverse, 5′‐TACAGGATTGTCGCCTTCAC‐3′; Human 18S: forward, 5′‐TCAAGAACGAAAGTCGGAGG‐5′ and reverse, 5′‐GGACATCTAAGGGCATCAC‐5′. Human 18S rRNA served as a control for the amount of cDNA present in each sample.

### 2.5 Immunostaining

For en face staining, the whole aorta was cut open longitudinally, permeabilized with 0.1% Triton X‐100 in PBS for overnight and blocked with 10% normal goat serum in Tris‐buffered saline containing 2.5% Tween‐20 for 24 hours at 4℃. Next, aortas were incubated with primary antibody in blocking buffer 48 hours at 4℃. After rinsing with washing solution (Tris‐buffered saline containing 2.5% Tween‐20) (4‐6 hours), fluorescence‐conjugated secondary antibodies were applied for 24 hours at 4℃. Finally, after another 3 rinses in the washing solution (4‐6 hours), aortas were mounted in the ProLong antifade reagent (Invitrogen). Aortas were examined by a laser‐scanning confocal microscope (FV‐1000 mounted on IX81, Olympus). For cell immunofluorescence staining, cells were fixed, permeabilized and incubated with antibody as indicated in figure legends followed by fluorescein‐conjugated secondary antibodies. F‐actin was stained with Alexa Fluorxi™ 488 Phalloidin (green) or Rhodamine Phalloidin (red). Cell nuclei were stained with DAPI.

### 2.6 Proteomics assay

HUVECs were lysed, and immunoprecipitation was performed with anti‐LXN antibody. LXN complex was resolved by SDS‐PAGE, and the bands were excised and digested with trypsin. Tryptic peptides were separated and analysed by LC‐MS/MS using a linear ion trap mass spectrometer (LTQ). The MS analysis was performed with one full MS scan followed by five MS/MS scans on the five most intense ions from the MS spectrum with the dynamic exclusion settings: repeat count 1, repeat duration 30 seconds and exclusion duration 30 seconds. MS/MS spectra were searched against the NCBInr_20130221 database using the Mascot algorithm (Version 2.3.02) with parent and fragment ion mass tolerance of 1.5 Da and 0.8 Da, respectively. Carbamidomethylation of cysteines and oxidation of methionines were allowed during the search of peptides. The maximum number of missed cleavages was set to 1, with trypsin as the protease.

### 2.7 Subcellular fractionation

Subcellular fractions were prepared by differential centrifugation of cell homogenates according to the protocol of cytosol and nuclear protein extraction kit (Beyotime Biotechnology).

### 2.8 Calpain activity assay

Cells were grown to confluence and lysed with radioimmunoprecipitation assay lysis buffer. The lysate was cleared of debris and used for the Calpain‐Glo protease assay (Promega) according to the manufacturer's protocol.

### 2.9 Statistical analyses

Data are expressed as means ± SEM. The statistical significance of differences was assessed by Student's *t* tests; a value of *P* < .05 was considered statistically significant.

## 3 RESULTS

### 3.1 Laminar shear stress down‐regulated LXN in ECs

**FIGURE 1:**
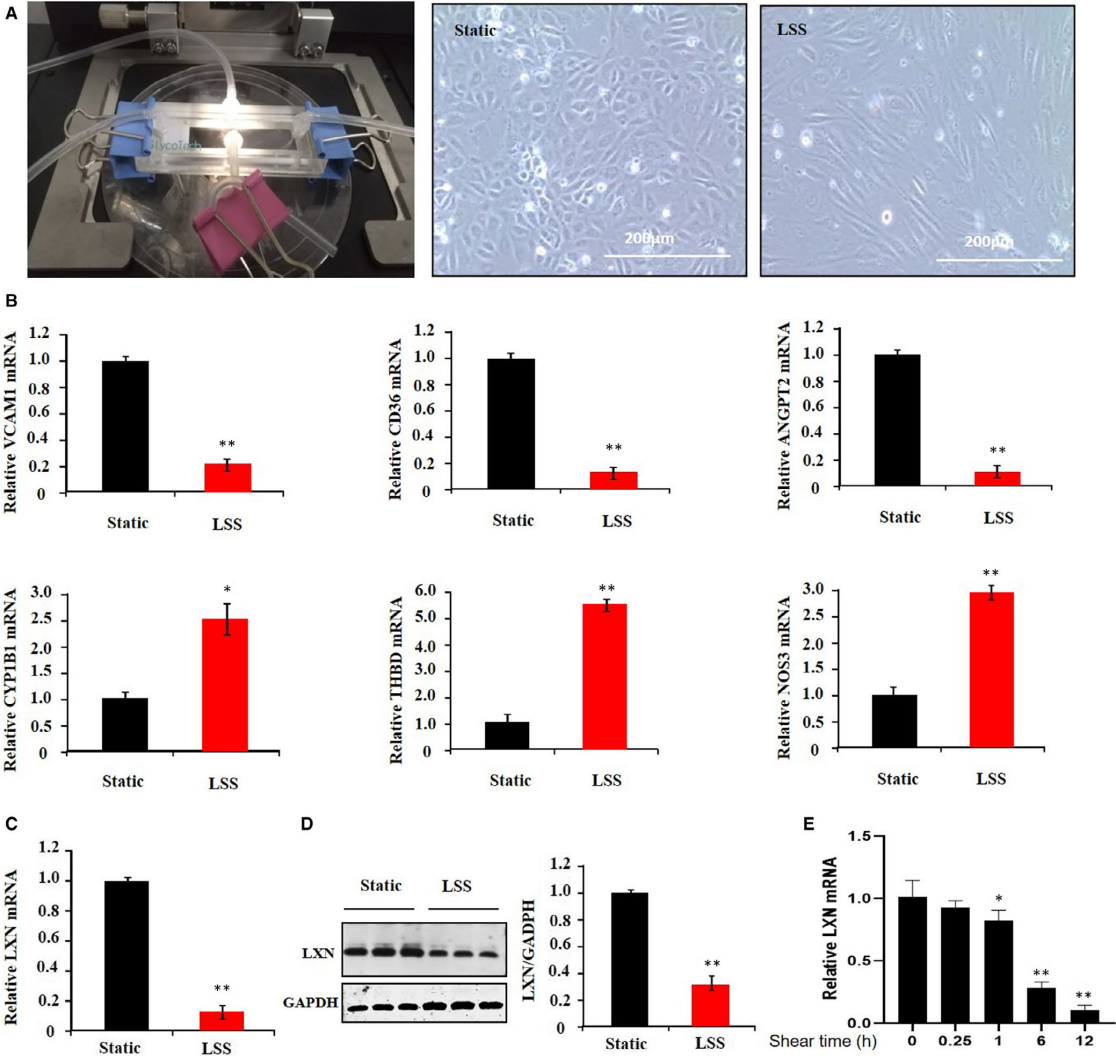
Laminar shear stress down‐regulates the expression of LXN in HUVECs. A, The parallel plate flow chamber comprised a polycarbonate plate attached to rectangular gaskets (Thickness: 0.010 in; Flow path width: 1.0 cm). HUVECs were cultured on a fibronectin coated slice. When cells grow to 90% confluence, attached the slice to flow chamber. The shear stress flow was initiated with EBM‐2 medium. Shear stress (15 dyne/cm^2^) was applied for 12 h. Cell morphology was demonstrated by Phase‐contrast photomicrograph. Scale bars = 200 μm. B, QPCR to determine the expression of gene as indicated. C and D, The expression of LXN was determined by Western blot and qPCR. E, HUVECs were exposed to LSS to detect the mRNA level of LXN at different time points. The results shown are the mean ± SEM. Data are representative of three independent experiments. *P < .05; **P < .01

HUVECs were subjected to laminar shear stress of 15 dynes/cm^2^ for 12 hours (Figure 1). As expected, we observed that laminar shear stress induces cell shape change (Figure 1A), up‐regulates vasoprotective genes (such as *CPY1B1, THBD* and *NOS3*) and, however, down‐regulates pro‐atherosclerosis genes (such as *VCAM1*, *CD36*, and *ANGPT2*) (Figure 1B). Interestingly, we found that laminar shear stress significantly inhibited the expression of LXN in HUVECs in time dependence manner (Figure 1C‐E), which prompted us to ask whether LXN involved in functional regulation of ECs.

### 3.2 *LXN* knockdown mimics LSS‐induced ECs shape change and cytoskeleton remodelling

**FIGURE 2:**
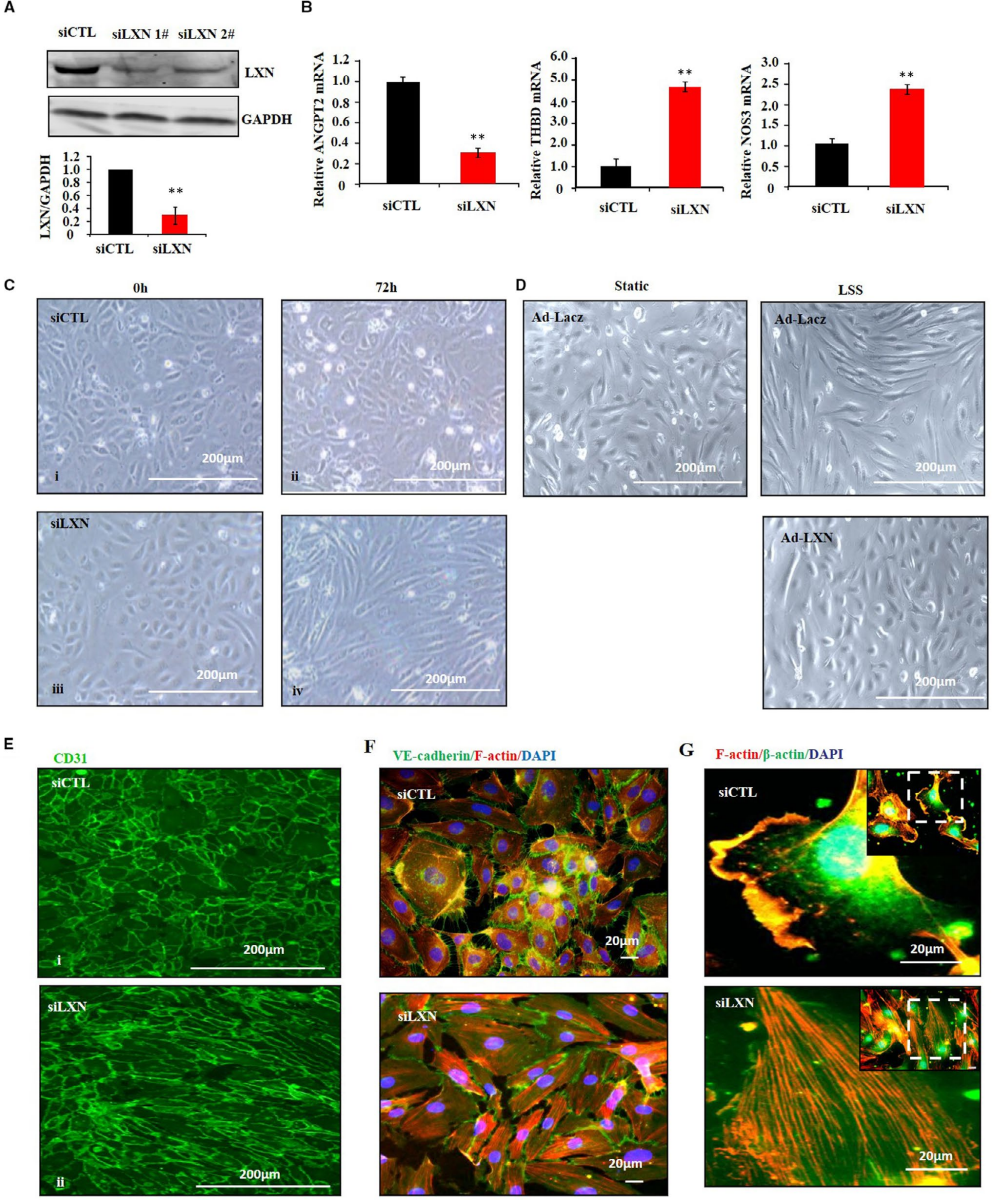
*LXN* knockdown regulates endothelial cell morphology and cytoskeleton remodelling. A, HUVECs were transfected with LXN siRNA for 72 h. The expression of LXN was determined by Western blot and qPCR. B, The expression of ANGPT2, THBD and NOS3 in *LXN* knockdown cell was determined by qPCR. C, Representative phase‐contrast microscopy images of HUVECs treated with siRNA (i, siCTL/0 h; ii, siCTL/72 h; iii, siLXN/0 h; iv, siLXN/72 h). D, HUVECs were infected with Ad‐LXN for 36 h, and then, the cells were treated by LSS (15 dyne/cm^2^) for 12 h. Cell morphology was demonstrated by Phase‐contrast photomicrograph. Scale bars = 200 μm. E, Representative immunofluorescence staining of HUVECs by CD31 (i, siCTL; ii, siLXN). F, siRNA‐treated HUVECs were stained with Phalloidin (red) and VE‐cadherin (green). G, siRNA‐treated HUVECs were stained with Phalloidin (red) and β‐actin (green). Nucleus was stained by DAPI

To explore the functional role of LXN in endothelial cell, function‐loss strategy was employed. Transfection HUVECs with *LXN* siRNA markedly inhibited endogenous LXN expression in HUVECs as determined by western blot (Figure 2A). QPCR result showed that *LXN* knockdown decreased *ANGPT2*, however, increased *THBD* and *NOS3* expression in HUVECs (Figure 2B), indicated the angio‐protective function of *LXN* knockdown in ECs. Interestingly, the morphologic change and cytoskeleton remodelling of cells were observed in *LXN* knockdown ECs (Figure 2C,E), similar to the morphological changes of ECs induced by laminar shear stress (Figure 1A). As shown in Figure 2C,E, the morphology of ECs transfected with CTL siRNA was randomly aligned and uniformly polygonal (Figure 2C‐i, ii; E‐i), whereas ECs transfected with *LXN* siRNA were elongated from the typical cobblestone pattern to uniformly fusiform aligned in the direction similar in appearance to the cells grown under laminar shear stress (Figure 2C‐iv, E‐ii), and the morphological changes of ECs induced by LSS could be reversed by overexpression of LXN (Figure 2D). We further investigate the effect of *LXN* loss on endothelial cell cytoskeleton remodelling by staining for F‐actin. As shown in Figure 2F,G, *LXN* knockdown resulted in the formation of actin stress fibres which running mainly in the longitudinal distribution of F‐actin filaments in the ECs (Figure 2F‐ii, G‐ii). Interestingly, we found that ECs display large and frequent lamellipodia formation in control conditions (Figure 2G‐i), and however, the lamellipodia formation were decreased in *LXN* knockdown cells (Figure 2G‐ii). Taken together, these data clearly demonstrate that LXN, at least, involves in endothelial cell shape change and actin cytoskeleton re‐organization in ECs.

### 3.3 LXN interacts with FLNA in ECs

**FIGURE 3:**
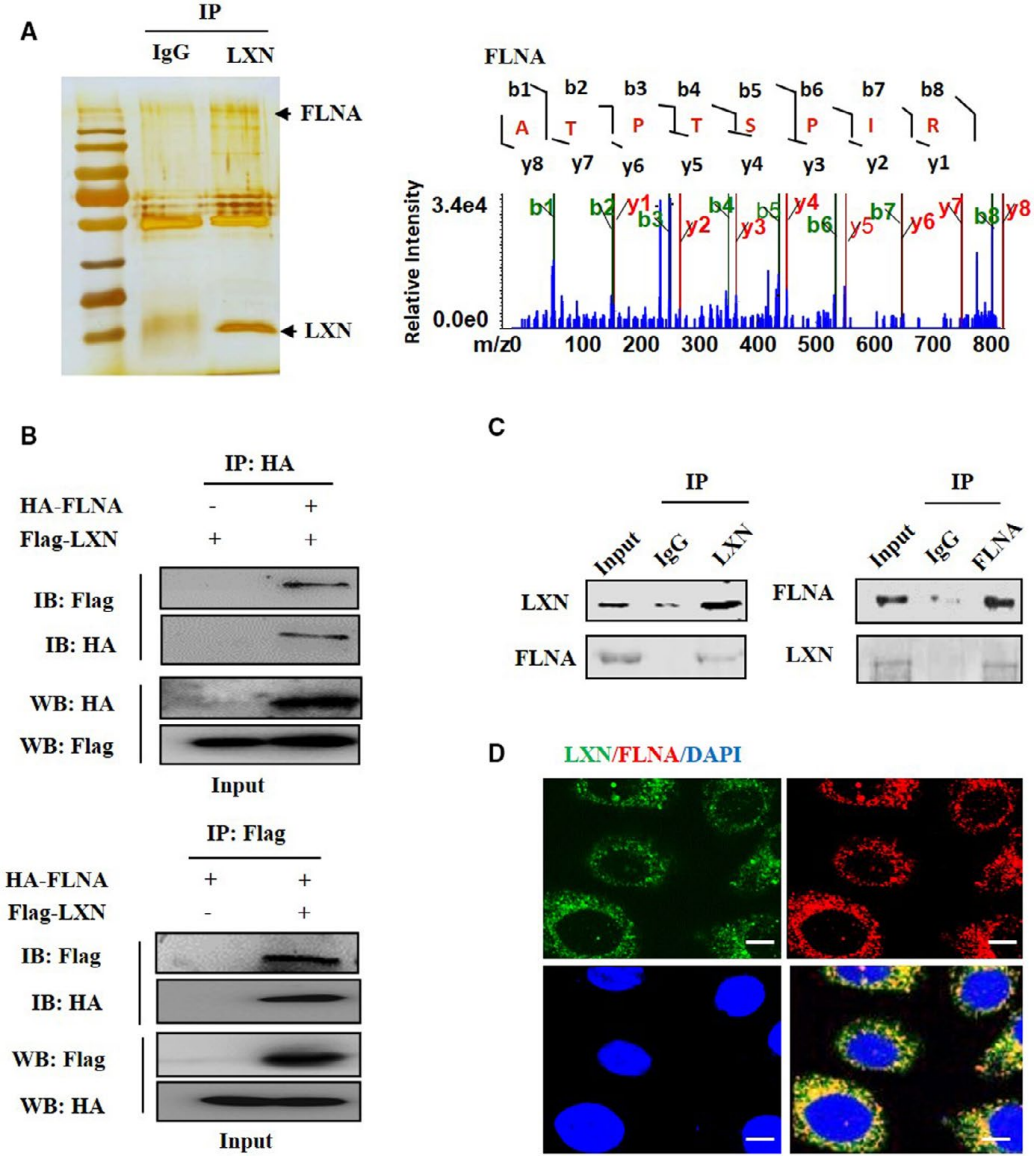
LXN interacts with FLNA. A, FLNA was identified as interacting protein with LXN. B, HA‐FLNA and Flag‐LXN expression vectors were transfected into 293T cells, and the interaction of LXN and FLNA was determined by co‐immunoprecipitation and Western blot (WB). C, Endogenous LXN forms a complex with interacts cytoskeleton proteins in HUVECs, as determined by immunoprecipitation (IP) with anti‐LXN antibody and separated by 10% SDS‐PAGE, followed by immunoblotting (IB) with antibody as indicated. D, Immunofluorescences show the co‐location of LXN and FLNA in HUVECs. Scale bars = 40 μm

To explore the mechanism how LXN regulates endothelial morphology, we have undertaken a proteomic screen to identify intracellular targets of LXN in ECs. To this end, HUVECs were lysed, and immunoprecipitation was performed with anti‐LXN antibody. LXN complex was resolved by SDS‐PAGE, and protein bands were cut into gel slices for trypsin digestion followed by liquid chromatography tandem mass spectrometry. FLNA, a scaffolding protein, was identified as a potential partner for LXN (Figure 3A). We further validated this interaction by immunoprecipitation and Western blot. As shown in Figure 3B, co‐immunoprecipitation experiments were performed in 293T cells transfected with HA‐FLNA and Flag‐LXN expression vectors, immunoprecipitation of HA‐FLNA led to co‐immunoprecipitation of Flag‐LXN when both proteins were co‐transfected. As a control, the anti‐HA antibody did not immunoprecipitate Flag‐LXN in the absence of HA‐FLNA. Similarly, immunoprecipitation of Flag‐LXN resulted in co‐immunoprecipitation of HA‐FLNA, whereas the anti‐Flag antibody did not immunoprecipitate HA‐FLNA in the absence of Flag‐LXN. To further confirm an in vivo interaction between LXN and FLNA, co‐immunoprecipitation and immunostaining assays were conducted. We validated that endogenous LXN exactly formed a physical complex with FLNA in HUVECs (Figure 3C). The interaction was further confirmed by immunostaining (Figure 3D). Together, our data strongly suggest that FLNA might be involved in binding to LXN and form a complex in ECs.

### 3.4 *LXN* knockdown regulates proteolytic cleavage of FLNA and promotes FLNA nucleus translocation in ECs

**FIGURE 4:**
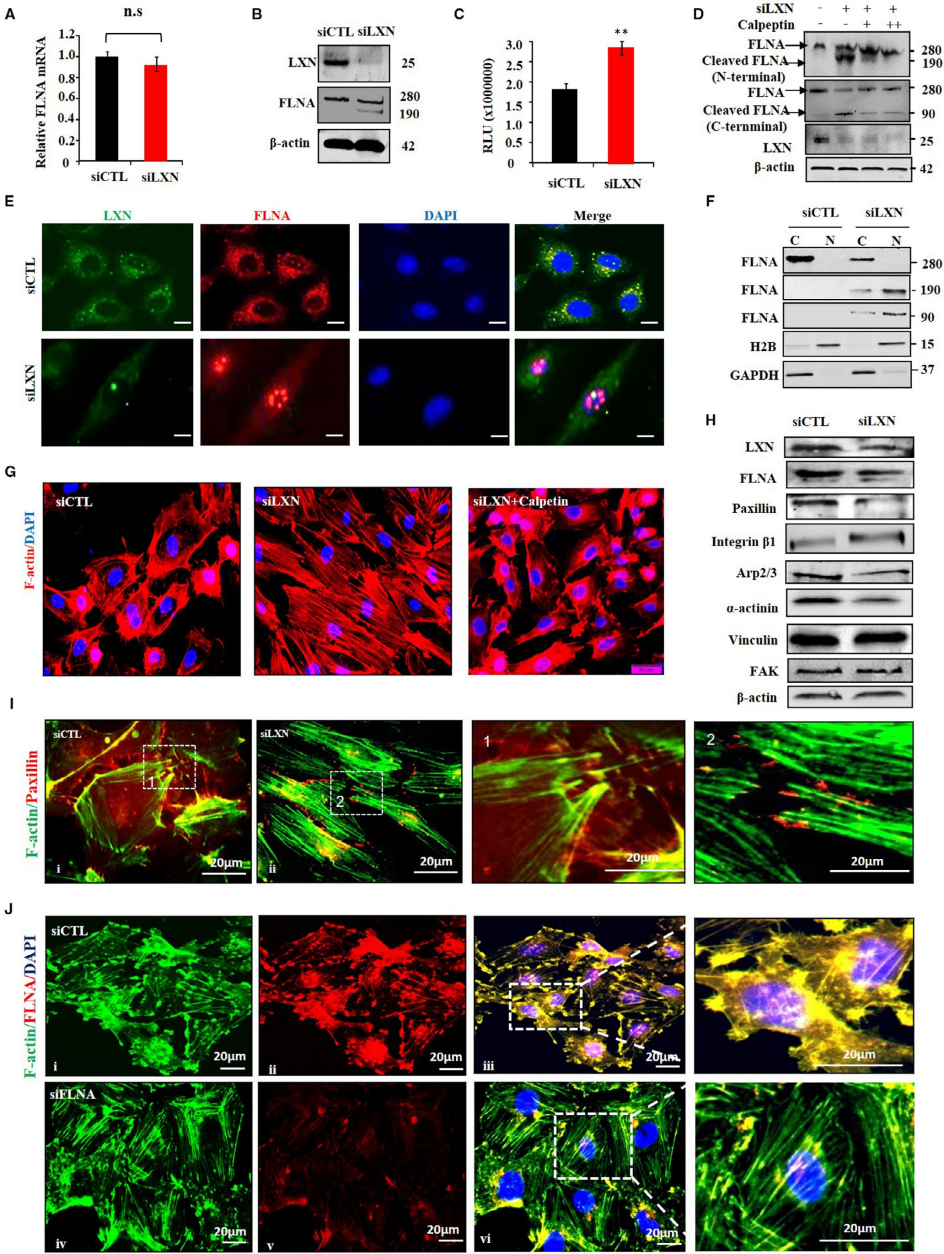
*LXN* knockdown modulates FLNA proteolytic cleavage and subcellular localization in ECs. A and B, HUVECs cultured in plates were treated with siCTL or siLXN for 72 h. The expression of FLNA was determined by qPCR (A) and Western blot (B). C, Control (siCTL) or LXN siRNA (siLXN) HUVECs were grown to confluence, and total lysates were used for the luminescent Calpain‐Glo protease assay. The relative luminescence units (RLU) were averaged and normalized to the amounts of proteins in cell lysates. D, HUVECs were transfected with siCTL or siLXN for 24 h, and then, cells were untreated or treated with calpeptin (10, 40 μg/mL) for 48 h. Cells were lysed and analysed by Western blot with antibodies as indicated. E, HUVECs cultured in plates were transfected with siCTL or siLXN for 72 h. Cells were washed, fixed, and immunofluorescence stained with anti‐LXN and anti‐FLNA antibody. Scale bars = 20 μm. F, HUVECs cultured in plates were treated with siCTL or siLXN for 72 h. The cytoplasmic (C) and nuclear (N) fractions of HUVECs were detected by Western blot with antibodies as indicated. G, HUVECs cultured in plates were pro‐treated calpeptin for 2 h and treated with siLXN for 72 h. HUVECs were stained with Phalloidin. Scale bars = 20 μm. H, Western blot shows the level of proteins as indicated in siCTL‐ and siLXN‐treated HUVECs. I, HUVECs cultured in plates were treated with siCTL or siLXN for 72 h. HUVECs were immunofluorescence stained with Phalloidin and anti‐paxillin antibody. High magnification images of the areas denoted with a dashed box were showed. Scale bars = 20 μm. J, Immunofluorescence staining shows the F‐actin associated with FLNA in HUVECs. siCTL or siFLNA‐treated HUVECs were immunofluorescence stained with Phalloidin and anti‐FLNA antibody. High magnification images of the areas denoted with a dashed box were showed. Scale bars = 20 μm

FilaminA, a well know F‐actin crosslink protein, which regulates signalling events involved in cell shape change and motility.^21^ Since LXN regulates cell shape, changing and actin cytoskeleton remodelling in ECs has been observed in our study (Figures 1 and 2). The interaction of LXN with FLNA promoted us to evaluate whether LXN effects FLNA protein levels in ECs. To test this hypothesis, LXN function‐loss was performed in ECs. As shown in Figure 4, we found that LXN knockdown did not affect the mRNA level of FLNA (Figure 4A), however, significantly decreased the protein level of FLNA (280 kD) in ECs (Figure 4B). Interestingly, we found a new band about 190 kD, which can also be recognized by FLNA antibodies, suggesting FLNA may be degraded (Figure 4B). FLNA is highly susceptible to proteolysis by calpain, and calpain‐induced cleavage products of 190 kD for FLNA have been reported.^22^ Therefore, we speculate whether LXN regulates calpain activity in endothelial cells. Using the luminescence Calpain‐Glo assay, we observed that calpain activity was increased in LXN knockdown ECs compared with control cells (Figure 4C). Therefore, we treated LXN knockdown ECs with calpeptin, a calpain inhibitor, FLNA and its fragments were determined by Western blot. We found LXN knockdown resulted in a decreasing of FLNA (280 kD), meanwhile increasing of cleaved FLNA (N‐terminal of FLNA,190 kD; C‐terminal of FLNA, 90 kDa), and however, this effect could be prevented by calpain inhibition (Figure 4D). It has been reported that full‐length (280 kDa) FLNA is mainly localized to the cytoplasm, the active cleaved form of FLNA localizes to the nucleus rather than the cytoplasm.^21^, ^23^ Therefore, we want to know whether LXN knockdown affects the full‐length and cleaved FLNA subcellular localization in ECs. We further determined the FLNA subcellular localization by immunostaining and Western blot. Immunostaining shows that FLNA is co‐localized with LXN in the cytoplasm, and LXN knockdown significantly promoted FLNA subcellular translocation from cytoplasm to nuclear in ECs (Figure 4E,F). Interestingly, calpain inhibitor can reverse the formation of stress fibres induced by LXN knockdown (Figure 4G). Taken together, our data clearly showed LXN maintains the stability of FLNA in cytoplasm, and LXN deficiency down‐regulates FLNA via calpain‐dependent pathway and promotes FLNA nucleus translocation.

### 3.5 LXN regulates cytoskeleton anchoring and focal adhesion formation through FLNA in ECs

FilaminA has been reported to involve in cytoskeleton remodelling and cytoskeleton anchoring.^24^, ^25^ We further asked whether LXN knockdown affects the stability of cytoskeleton proteins. We first tested some cytoskeleton related proteins, such as integrin‐β, paxillin, Arp2/3, α‐actinin, FAK and vinculin in HUVECs treated with siCTL or siLXN. Western blot showed that the expression of integrin‐β increased slightly after knocking down LXN, while the expression of paxillin, Arp2/3 and α‐actinin was decreased significantly (Figure 4H). Because paxillin has been reported to implicate in the formation of focal adhesion (FA) and cytoskeleton anchoring, we next investigated whether FLNA has effect on cytoskeleton anchoring in ECs. To this end, control or LXN knockdown cells were fixed, and paxillin and F‐actin were immunofluorescently probed by using confocal microscopy. As shown in Figure 4I,F‐actin filaments, which are randomly aligned in control cells, are linked to focal adhesions at their ends (Figure 4I‐i). However, in LXN knockdown cells, the association between actin filaments and focal adhesion was disrupted with the disappearance of focal adhesion such as structures and thus resulted in the distribution of F‐actin filaments in longitudinal direction of the cell, as well as the morphologic change (Figure 4I‐ii). We next knockdown FLNA in ECs (Figure 4J). We found that the focal adhesion such as plaques was impaired and resulted in the formation of actin stress fibres which running mainly in the longitudinal distribution of F‐actin filaments in the ECs (Figure 4J‐vi), which indicating decrease of FLNA in ECs contributes to the formation of stress fibres. Taken together, our data clearly showed functional role of LXN, as a protein complex with FLNA, involved in the regulatory of focal adhesion formation and anchoring with actin filaments in ECs.

### 3.6 *LXN* deficiency modulates endothelial cell shape change and improves vascular function in mice

**FIGURE 5:**
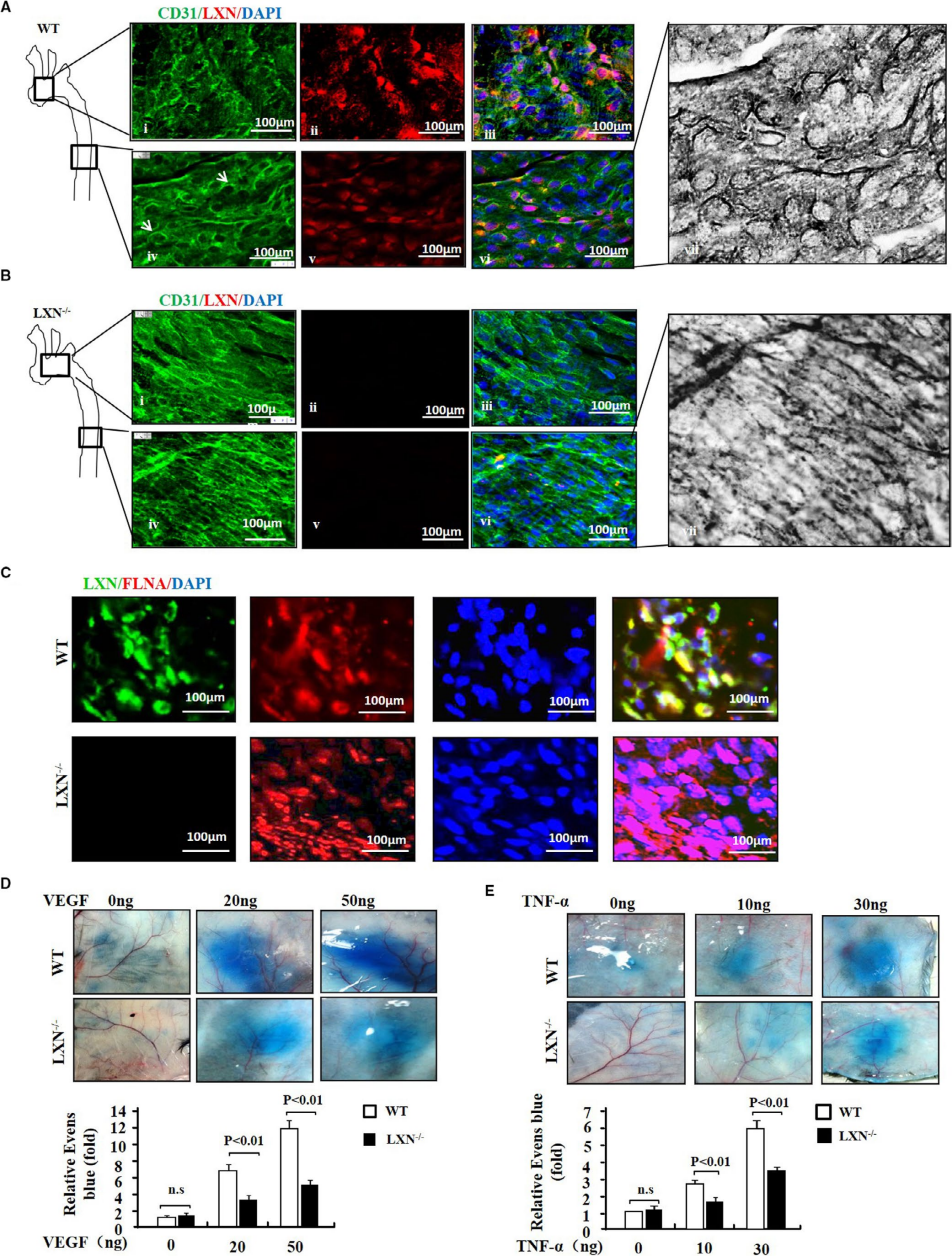
*LXN* deficiency improves vascular function in mice. A and B, en face immunostaining shows the ECs morphology in aortic arch (i‐iii) or thoracic aorta (iv‐vi) in WT (A) and LXN^‐/‐^ (B) mice. ECs were stained with CD31 and LXN antibody. Scale bars = 100 μm. C, en face immunostaining showed the localization of LXN and FLNA in ECs of thoracic aorta of WT and LXN^‐/‐^ mice. D and E, Vascular permeability induced by VEGF (D) and TNF‐α (E) in Miles assay with different doses in the skin of WT and LXN^‐/‐^ mice. Quantification of VEGF or TNF‐α‐induced Evans blue (EB) incorporation in the skin (n = 6). The results shown are the mean ± SEM

To evaluate the role of LXN in regulating endothelial cell morphology in vivo, we examined the cell shape change and distribution of cytoskeleton using enface staining of endothelial cell lining the aorta arch of WT and *LXN* ^‐/‐^ mice. Confocal microscopy revealed that LXN exists in the endothelium layer in aortic (Figure 5). The level of LXN in the endothelium of the aortic arch was significantly higher than that in the thoracic aorta in WT mice (Figure 5A‐ii,v‐i, iv), CD31 staining showed that the cell displayed a polygonal shape (Figure 5A‐i,iv). However, in *LXN* ^‐/‐^ mice, the endothelial cells were elongated and displayed fusiform shape (Figure 5B‐i, iv). FLNA was found in both the perinuclear and the cytoplasm of the endothelial cells in WT mice, and the colocalization of LXN and FLNA was observed (Figure 5C). However, FLNA was observed to transfer to the nucleus of the endothelial cells in LXN^‐/‐^ mice. This is consistent with the results observed in the cells in vitro (Figure 4E). Together, these results showed that LXN modulates endothelial cell shape change and actin cytoskeleton re‐organization in vivo.

Next, we evaluated the effect of LXN deficiency on vascular function in vivo. We first performed the miles assay in WT and *LXN* ^‐/‐^ mice by Evans blue injecting. Vascular permeability within the aorta was assessed by measuring leakage of Evans blue dye into the vascular wall. We found that VEGF (Figure 5D) and TNF‐α (Figure 5E) stimuli significantly increased the Evans blue dye visibility in flank skin in WT mice. However, Evans blue dye leakage induced by VEGF or TNF‐α was significantly reduced in *LXN* ^‐/‐^ mice. These data clearly indicate that *LXN* knockout significantly attenuates microvessel permeability.

**FIGURE 6:**
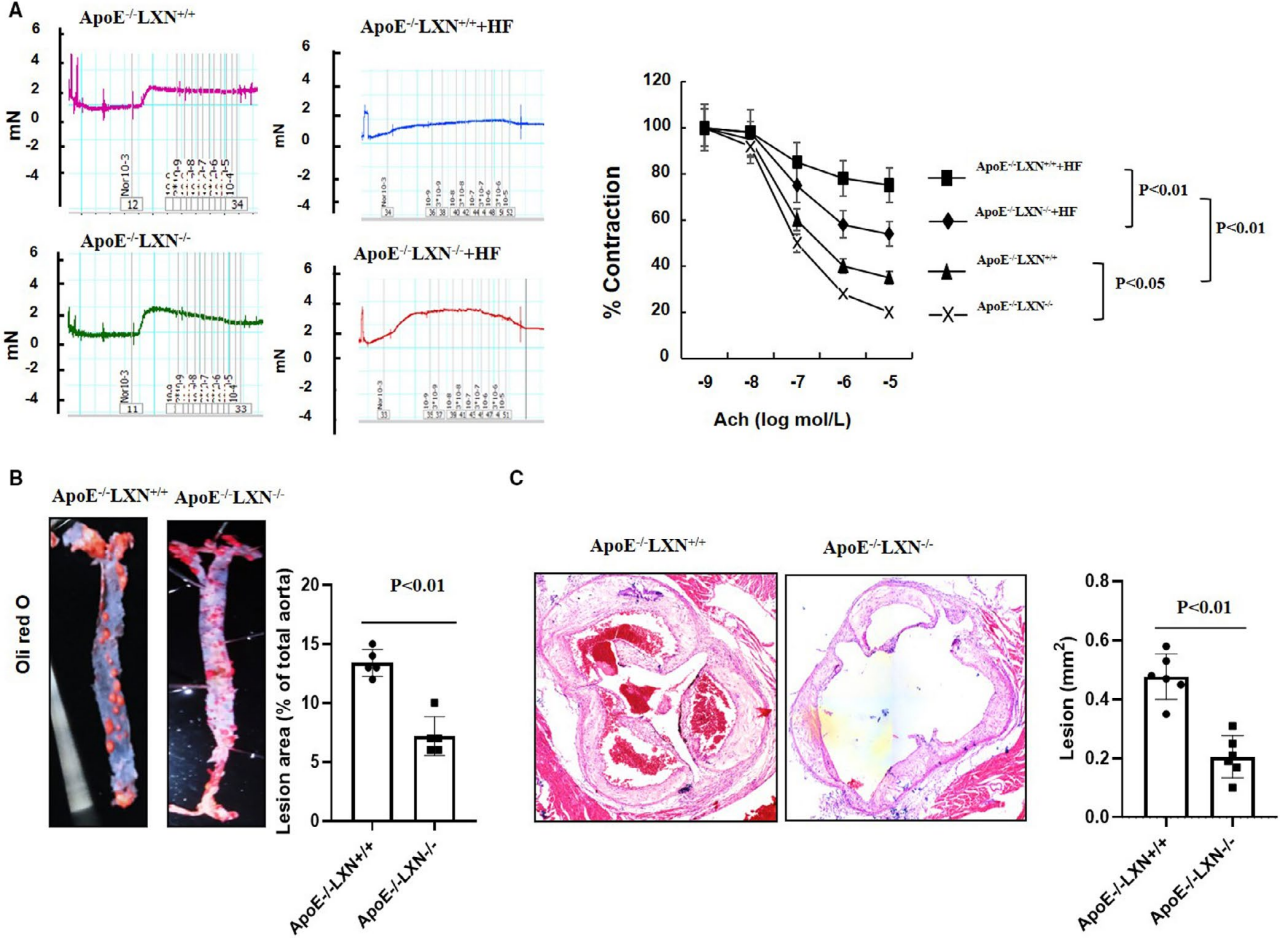
*LXN* deficiency improves vasodilation capacity and atherosclerosis in HF‐induced ApoE^‐/‐^ mice. A, Relaxation responses to the cumulative addition of Ach for aorta arteries contracted with 1µm NE for ApoE^‐/‐^ LXN^+/+^ and ApoE^‐/‐^ LXN^‐/‐^ mice feed with high‐fat diet (n = 4). B, Representative images of Oil Red O staining of en face preparations of aortas and quantification of the atherosclerotic surface area of the entire aorta of ApoE^‐/‐^ LXN^+/+^ and ApoE^‐/‐^ LXN^‐/‐^ mice (n = 6). C, HE staining of aortic arch root of ApoE^‐/‐^ LXN^+/+^ and ApoE^‐/‐^ LXN^‐/‐^ mice (n = 6). The results shown are the mean ± SEM

We further evaluated the vascular protective role of LXN deficiency in atherosclerosis. We constructed *ApoE* ^‐/‐^ *LXN* ^‐/‐^ double knockout mice, and the atherosclerosis model was established by feed the mice with high‐fat diet for 16 weeks. Analysis of vasodilation capacity showed that high‐fat diet greatly impaired vasodilation function. *LXN* deficiency not only reverses the dysfunction of vasodilation caused by high‐fat diet, but also significantly improves the vasodilation in normal mice (Figure 6A), further indicating the protective effect of LXN deficiency on blood vessels. Finally, and most importantly, we found that *LXN* deficiency significantly inhibited the formation of atherosclerotic plaques induced by high‐fat diet (Figure 6B,C). Taken together, these results clearly show that *LXN* deficiency can improve vascular function and alleviate the formation of atherosclerotic plaque in mice.

## 4 DISCUSSION

**FIGURE 7:**
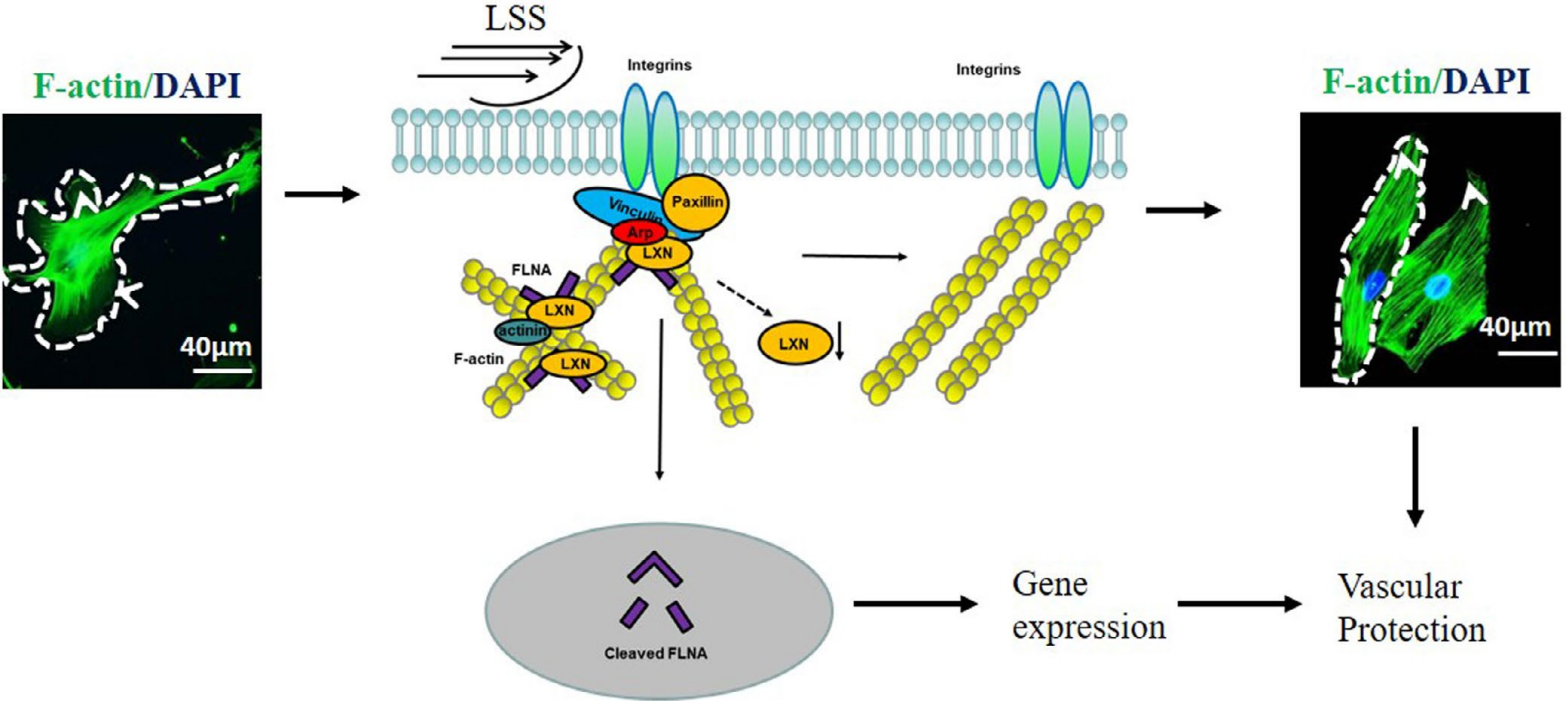
Schematic presentation of the cell shape and stress fibre network in static and laminar shear stress or LXN deficiency ECs. In normal condition, transverse arcs are generated from Arp2/3, α‐actinin and FLNA crosslinked actin filaments in normal ECs. Under laminar shear stress, LXN loss resulted in the proteolytic cleavage and subcellular localization of FLNA, down‐regulation of Arp2/3, α‐actinin and paxillin in ECs. As a result, ECs were elongated from the typical cobblestone pattern to uniformly fusiform and showed the distribution of F‐actin filaments in longitudinal direction of the cell

In the present study, we discovery a novel function of LXN in ECs. We demonstrate an interesting phenomenon that laminar shear stress induces the down‐regulation of LXN. Conversely, *LXN* knockdown could mimic laminar shear stress induced cell morphologic change in ECs. Proteomic reveals that LXN forms a complex with FLNA, and *LXN* knockdown enhances proteolytic cleavage of FLNA and subcellular localization, and thus involving regulatory of EC morphologic change and cytoskeleton remodelling (Figure 7). Animal models show that *LXN* deficiency significantly improves vascular permeability, vasodilation and atherosclerosis in mice.

Vascular ECs, which line the inner surface of blood vessels, are important in maintaining structural and functional roles of blood vessels.^26^ It is well known that ECs are continuously exposed to shear stress in vivo, and the ECs respond to shear stress by changing their morphology and function, which are important for maintaining cellular homeostasis in vascular system.^26^, ^27^, ^28^, ^29^ In this regard, two types of shear stress were reported in large vessel: one is LSS, which is vascular protective and founded in straight arterial regions; and the other is DSS, which is vascular harmful and founded in branched or curved regions.^29^, ^30^, ^31^ LSS induces vascular ECs morphologic change has been reported, and these morphologic changes are accompanied by cytoskeleton remodelling, as well as actin filaments becoming rearranged into bundles of stress fibres and aligned in the direction of the shear stress.^5^, ^32^ We found that LXN was highly expressed in ECs and down‐regulated by LSS (Figure 1). Interestingly, we observed that *LXN* knockdown resulted in the morphologic change and F‐actin filaments remodelling just like what LSS does in ECs (Figure 2). In this regard, our findings provided compelling evidence, demonstrating that LXN might be a novel molecular in response to shear stress of ECs.

To reveal the mechanism underline LXN regulating endothelial cell morphology, we have undertaken a proteomic screen to identify intracellular targets of LXN in ECs, and FLNA was identified as partner of LXN in ECs (Figure 3). FLNA is a 280 kDa dime with two N‐terminal actin‐binding domains.^33^ Dimerization of FLNA forms V‐shaped molecules that crosslink actin filaments into orthogonal networks and maintains endothelial cell morphology.^34^ A large number of literatures have reported that FLNA plays a key role in the cytoskeleton and determines cell shape and locomotion.^21^, ^35^ Indeed, FLNs can be regulated through cleavages by calpains.^22^ It has been reported that FLNA can be cleaved by calpain proteases, generating 190‐ and 90‐kDa fragments.^36^ Importantly, FLNA fragments can be transferred to the nucleus to regulate gene expression.^21^, ^23^ We further showed that *LXN* knockdown leads to activation of calpain (Figure 4C), and results in FLNA degradation and subcellular translocation (Figure 4D‐F). It was important that siLXN‐induced FLNA degradation and stress fibre formation could be inhibited by calpain specific inhibitors (Figure 4D and G). These results suggest that LXN can maintain the stability of FLNA in the cytoplasm in ECs, thus maintaining normal ECs morphology. In addition, filamin has been shown to interact with integrin β cytoplasmic domain constructs and depletion of filamin results in integrin activation.^37^ We found that LXN knockdown up‐regulates the protein level of integrin β1, and however, destroys protein level of cytoskeleton proteins as paxillin, Arp2/3 and α‐actinin (Figure 4H). Arp2/3 is very important for maintenance of cell morphology.^24^, ^25^ Activation of the Arp 2/3 complex could promote the formation of branched actin networks, especially, the formation of transverse arcs in lamellipodia area by combining short myosin filaments and actin filaments.^38^, ^39^ Alpha‐actinin is a cytoskeletal actin‐binding protein that crosslinks actin filaments by forming an anti‐parallel rod shaped dimer.^40^ Functionally, α‐actinin plays multiple important roles in the cell involved in linking the cytoskeleton to transmembrane proteins, regulating the activity of receptors and serving as a scaffold to connect the cytoskeleton to diverse signalling pathways.^40^ We found that *LXN* loss could induce the parallel distribution of F‐actin stress fibres in ECs (Figure 2), as well as down‐regulation of α‐actinin (Figure 4H). Together, these data support our hypothesis that LXN is involved in ECs morphologic change via the regulation of cytoskeleton remodelling.

Finally, and most importantly, our results show that LXN deficiency has protective effects on vascular homeostasis. Disturbed shear stress, hypertension and diabetes can damage vascular endothelium, leading to the occurrence and development of atherosclerosis.^41^, ^42^, ^43^ Laminar shear stress has been reported to improve vascular function, including reducing endothelial permeability, increasing vasodilation and the expression of cytoprotective genes.^44^, ^45^ However, laminar shear seems to be a necessary condition for endothelial cell integrity, and laminar shear stress is a ‘survival’ factor rather than a ‘growth’ factor of endothelial cells.^3^ In animal models, we demonstrate that *LXN* deficiency inhibits VEGF and TNF‐α‐induced vascular permeability (Figure 5D,E), as well as significantly improves vasodilation and atherosclerosis in mice fed with high‐fat diet (Figure 6).

In summary, our results suggest that LXN deficiency has a protective effect on blood vessels. Our data support LXN as an important regulator of ECs morphology through forming a complex with cytoskeleton proteins. LXN directly interacts with FLNA in vascular ECs, and this interaction is functional, because LXN loss enhances FLNA proteolytic cleavage and subcellular localization; and it is likely physiological, as it suppresses actin filaments anchoring with focal adhesion and results in cell morphologic change which is similar with that of laminar shear stress does on ECs. Importantly, we show that *LXN* deficiency significantly improves vascular permeability, vasodilation and atherosclerosis in mice, which indicated LXN deficiency has a protective effect on vascular homeostasis, and provides new targets for the treatment of vascular diseases.

## CONFLICT OF INTERESTS

The authors declare no competing financial interests.

## AUTHOR CONTRIBUTION

**Guozhang He:** Data curation (equal); Investigation (equal); Methodology (equal); Writing‐original draft (equal). **Shuang Kan:** Data curation (equal); Investigation (equal); Validation (equal). **Shaohua Xu:** Investigation (equal); Methodology (equal); Writing‐original draft (equal). **Xuchen Sun:** Data curation (equal); Investigation (equal); Resources (equal). **Rong Li:** Investigation (equal); Resources (equal). **Wei Shu:** Investigation (equal); Writing‐original draft (equal). **Ming Chen:** Conceptualization (equal); Funding acquisition (equal); Investigation (equal); Project administration (equal); Resources (equal); Supervision (equal); Validation (equal); Writing‐original draft (equal).

## Data Availability

The data that support the findings of this study are available from the corresponding author upon reasonable request.
